# Prevalence of *crt* and *mdr*-*1* mutations in *Plasmodium falciparum* isolates from Grande Comore island after withdrawal of chloroquine

**DOI:** 10.1186/s12936-016-1474-4

**Published:** 2016-08-15

**Authors:** Bo Huang, Qi Wang, Changsheng Deng, Jianhua Wang, Tao Yang, Shiguang Huang, Xin-zhuan Su, Yajun Liu, Longhua Pan, Guoming Li, Di Li, Hongying Zhang, Afane Bacar, Kamal Said Abdallah, Rachad Attoumane, Ahamada M. S. A. Mliva, Shaoqin Zheng, Qin Xu, Fangli Lu, Yezhi Guan, Jianping Song

**Affiliations:** 1 Science and Technology Park, Guangzhou University of Chinese Medicine, Guangzhou, 510006 Guangdong People’s Republic of China; 2 School of Medicine, Jinan University, Guangzhou, 510632 Guangdong People’s Republic of China; 3 Laboratory of Malaria and Vector Research, National Institute of Allergy and Infectious Diseases, National Institutes of Health, Bethesda, MD 20892 USA; 4 State Key Laboratory of Cellular Stress Biology, School of Life Sciences, Xiamen University, Xiamen, 361005 Fujian People’s Republic of China; 5 The First Affiliated Hospital, Guangzhou University of Chinese Medicine, Guangzhou, 510006 Guangdong People’s Republic of China; 6 Guangdong Newsouth Artepharm Co., Ltd, Guangzhou, 510405 Guangdong People’s Republic of China; 7 National Malaria Control Programme, BP 500 Moroni, Union of Comoros; 8 Ministry of Health Comoros, BP 403 Moroni, Union of Comoros; 9 Research Institute of Tropical Medicine, Guangzhou University of Chinese Medicine, Guangzhou, 510006 Guangdong People’s Republic of China; 10 Department of Parasitology, Zhongshan School of Medicine, Sun Yat-sen University, Guangzhou, 510080 Guangdong People’s Republic of China

**Keywords:** Comoros, *Plasmodium falciparum*, Chloroquine resistance, *pfcrt*, *pfmdr*-*1*

## Abstract

**Background:**

In Comoros, the widespread of chloroquine (CQ)-resistant *Plasmodium falciparum* populations was a major obstacle to malaria control, which led to the official withdrawal of CQ in 2004. Continuous monitoring of CQ-resistant markers of the *P. falciparum* CQ resistant transporter (*pfcrt*) and the *P. falciparum multiple drug resistance 1* (*pfmdr*-*1*) is necessary inder to obtain first-hand information on CQ susceptibility of parasite populations in the field. The objective of this study is to assess the prevalence and evolution of CQ-resistance in the *P. falciparum* populations on the Comoros’ Grande Comore island after withdrawal of CQ.

**Methods:**

A total of 207 *P. falciparum* clinical isolates were collected from the island, including 118 samples from 2006 to 2007 and 89 samples from 2013 to 2014. Nucleotide substitutions in the *pfcrt* and *pfmdr*-*1* genes linked to CQ response in parasite isolates were assessed using nested PCR and DNA sequencing.

**Results:**

From the *pfcrt* gene segment sequenced, we detected C72S, M74I, N75E, and K76T substitutions in the parasite isolates collected from both 2006**–**2007 to 2013**–**2014 periods. Significant decline of *pfcrt* resistant alleles at C72S (42.6 to 6.9 %), M74I (39.1 to 14.9 %), N75E (63.5 to 18.3 %), and K76T (72.2 to 19.5 %) from 2006–2007 to 2013–2014 were observed, and the frequency of *pfcrt* wild type allele was significantly increased from 19.1 % in 2006–2007 to 75.8 % in 2013–2014. Sequence analysis of *pfmdr*-*1* also detected point mutations at codons N86Y, Y184F, and D1246Y, but not S1034C and N1042D, in the isolates collected from both examined periods. An increasing trend in the prevalence of the *pfmdr*-*1* wild type allele (NYD, 4.3 % in 2006–2007; and 28.7 % in 2013–2014), and a decreasing trend for *pfmdr*-*1* N86Y mutation (87.0 % in 2006–2007; and 40.2 % in 2013–2014) were observed in our samples.

**Conclusions:**

The present data indicate that the prevalence and patterns of mutant *pfcrt* and *pfmdr*-*1* dramatically decreased in the Grande Comore isolates from 2006 to 2014, suggesting that the CQ-sensitive *P. falciparum* strains have returned after the withdrawal of CQ. The data also suggests that the parasites with wild type *pfcrt*/*pfdmr*-*1* genes may have growth and/or transmission advantages over the mutant parasites. The information obtained from this study will be useful for developing and updating anti-malarial treatment policy in Grande Comore island.

## Background

Despite being a readily preventable and treatable disease, malaria still infects more than 210 million people and kills approximately 438,000 individuals annually [1]. In the Union of Comoros (including Moheli, Anjouan Island, and Grande Comore islands), *Plasmodium falciparum* infection was one of the most serious public health problems until 2013, and malaria made up 15–30 % of the hospitalization cases and contributed 15–20 % of registered deaths in the pediatric services [2]. One of the main factors contributing to the disease burden is the emergence and spread of parasites resistant to anti-malarial drugs in malaria-endemic areas of the world [3]. Chloroquine (CQ) has been the first-line treatment of acute uncomplicated *falciparum* malaria in this island nation since the 1950s. Unfortunately, the first case of CQ-resistance (CQR) *P. falciparum* malaria was reported in Comoros in 1980 [4]. Since the first report, various studies have subsequently confirmed that the emergence and spread of CQR parasite strains [5–7], leading to the replacement of CQ with artemisinin-based combined therapy (ACT), including artemether-lumefantrine (AL), as the first-line therapy for uncomplicated *P. falciparum* malaria in 2004. However, it should be noted that there was period (between 2004 and 2007) with overlapping CQ and AL treatments due to the unavailability of AL treatment in some health facilities in Comoros. Additionally, massive application of long-lasting insecticide-treated nets and indoor residual sprayings had been implemented in Comoros since 2007. Furthermore, mass drug administration (MDA) with a therapeutic dose of artemisinin-piperaquine (AP) plus a low-dose of primaquine (APP, Artepharm Co. Ltd, PR China) was launched in 2007, 2012, and 2013 on Moheli, Anjouan, and Grande Comore islands, respectively. According to a report from the Ministry of Health, the numbers of annual malaria cases have been dramatically reduced after MDA, from 108,260 in 2006–2154 in 2014 in Union of Comoros (a 97.7 % reduction) and from 92,480 in 2006–2142 in 2014 in Grande Comore (a 98.0 % reduction). The dramatic reduction in annual malaria cases in Grande Comore could be mainly attributed to ACT-based MDA regimens in synergy with other malaria control measures. Currently, delayed parasite clearance (DPC) after ACT treatment has been reported in countries of Southeast Asia, including Cambodia, Thailand, Myanmar, Vietnam, and Laos [8–10]. The increased K13-propeller gene mutations previously associated with DPC among *P. falciparum* populations from 2013 to 2014 in Grande Comore (a ~20 % increment) may present new challenges in the ACT efficacy in the future [11, 12]. To achieve the ambitious goal to completely eliminate malaria by 2020 in Comoros, as well as to preserve the high efficacy of ACT, there is an urgent need to develop and update anti-malarial guidance in Comoros.

Resistance to CQ in *P. falciparum* parasites is mainly linked to mutations in the *P. falciparum* CQR transporter gene (*pfcrt*), which encodes a protein localized at the membrane of parasite digestive vacuole (DV). A second gene called *P. falciparum* multidrug resistance gene 1 (*pfmdr*-*1*) that encodes a P-glycoprotein homologue and is also located at the membrane of the parasite DV may modulate the level of resistance. Specific mutations in *pfcrt* (K76T) and *pfmdr*-*1* (N86Y) have been used as molecular markers for monitoring CQR in the field parasite populations [13, 14]. The continuous monitoring of molecular markers is a very useful tool for malaria control in endemic areas, since it may detect changes in parasite susceptibility to anti-malarial drugs and provide guidance to therapeutic policies. Several studies have assessed allele frequencies in different time periods in various malaria-endemic areas. For example, in Malawi over 8 years [15], in Papua New Guinea over 12 years [16], in Gabon over 14 and 25 years [17, 18], and in Eastern Kenya over 14 years [19]. In Malawi [15, 20, 21], Gambia [18], Kenya [19], Ethiopia [22], and Tanzania [23], the withdrawal of CQ chemotherapy led to a decline in the frequency of mutant *pfcrt* and *pfmdr*-*1* alleles and the return of CQ sensitive (CQS) malaria. In the contrary, high frequencies of CQR strains were still detected in some endemic regions despite the withdrawal of CQ from national treatment guidelines [17, 24, 25].

Before the change of the drug policy in Comoros, clinical failure rates of CQ therapy ranged between 31 and 90 % [7, 26, 27]. Subsequent genotyping showed that the prevalence of the *pfcrt* K76T and the *pfmdr*-*1* N86Y mutations ranged from 62 to 98 %, and from 90 to 100 %, respectively [7, 26, 28]. After the change in the drug policy, the frequency of the 76T and 86Y mutant alleles were 43–80 and 97–99 %, respectively, in 2006 [29–31]. Currently, the status of *P. falciparum* susceptibility to CQ in Comoros is unknown. Monitoring the parasite’s CQ susceptibility is crucial for considering the possibility of reintroducing this safe and affordable drug in Comoros. Thus, the aim of this study is to assess the prevalence and evolution of CQR by investigating the temporal variations of *pfcrt* and *pfmdr*-*1* gene polymorphisms in *P. falciparum* isolates collected from Grande Comore during the periods of 2006–2007 and 2013–2014.

## Methods

### Study sites

**Fig. 1:**
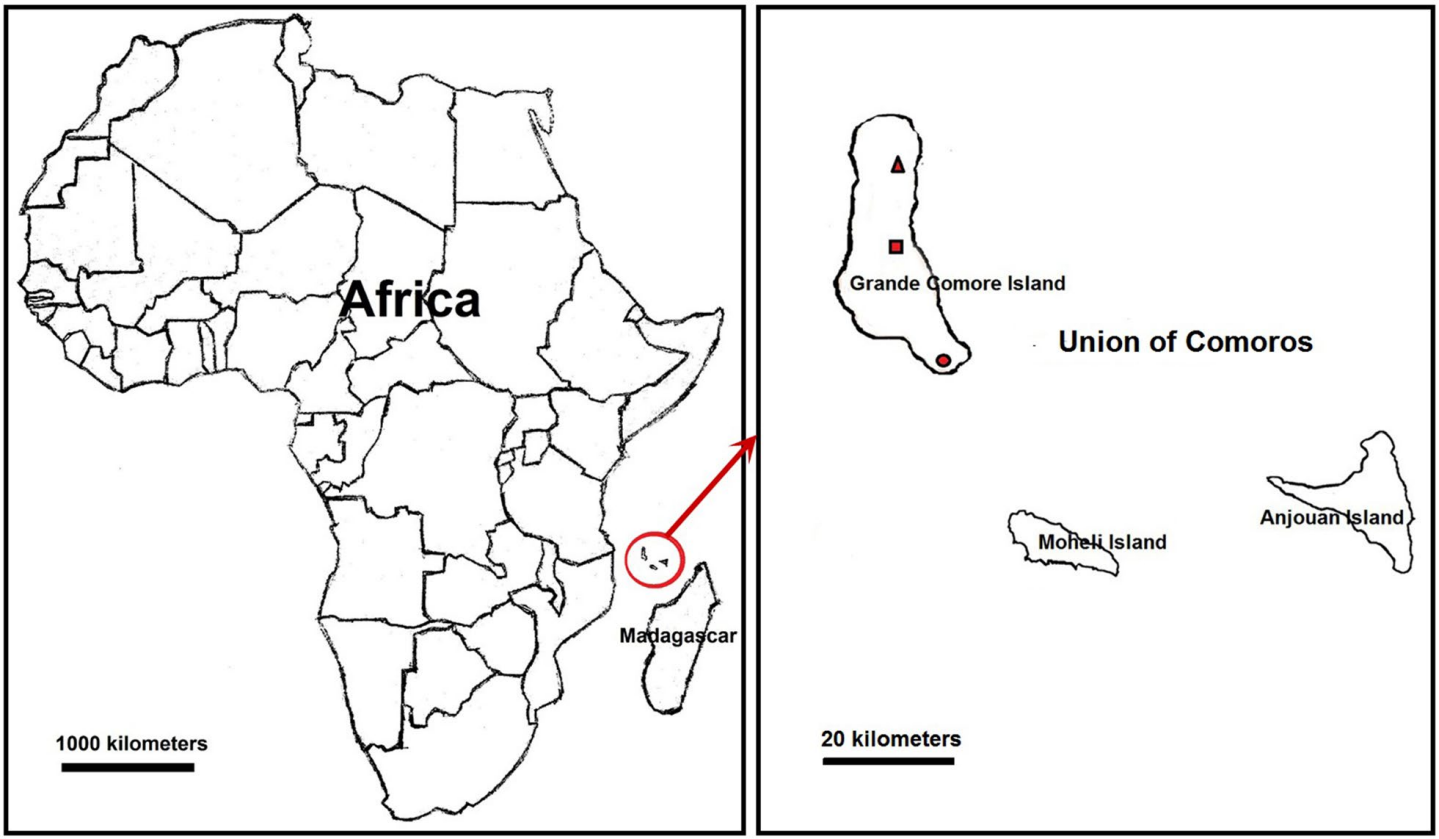
Map of Grande Comore island, Union of Comoros showing the locations of Mitsoudje Center Hospital (*open triangle*), National Malaria Center (*open square*), and Mitsamiouli Center Hospital (*open circle*) where *P. falciparum* isolates were collected

This study was conducted in three malaria endemic sites of the Grande Comore Island (Mitsoudje Centre Hospital, National Malaria Centre, and Mitsamiouli Centre Hospital), Union of Comoros (Fig. 1). The island is located in the Indian Ocean between Madagascar and the eastern coast of Africa, with 11°00′–12°00′S latitude and 43°10′–43°35′E longitude. The population of this island was estimated to be 420,000 in 2012. The island has a tropical climate with temperature ranging from 11 to 35 °C and rainfall of 1000–3000 mm per year (rainy season between November and April and dry season between May and October). Malaria is transmitted principally by *Anopheles gambiae s.l.* and *Anopheles funestus* [2]. Malaria transmission is year-round with a peak of transmission during the rainy season. *P. falciparum* and *Plasmodium malariae* malaria are present on the island, with *P. falciparum* being the predominant species (>95.5 %).

### Study samples

Ethical approval for this study was obtained from the Ethics Committees of Comoros Ministry of Health (No. 07-123/VP-MSSPG/DNS) and Guangzhou University of Chinese Medicine (No. 2012L0816). Blood samples were collected in two different periods (March 2006–October 2007, and March 2013–December 2014) from patients with symptomatic malaria admitted to Mitsoudje Center Hospital, National Malaria Center, and Mitsamiouli Center Hospital for anti-malarial drug treatment. Written informed consent from all adults or legal guardians of children was also obtained. Inclusion criteria were patients infected with *P. falciparum*, but not other human malaria species, as confirmed by peripheral thick and thin blood smear examination after Giemsa staining. A 1.0 ml of whole blood was collected from *P. falciparum* patients in an EDTA tube and stored at −20 °C. A total of 207 *P. falciparu*m clinical blood samples were collected, including 118 samples from March 2006 to October 2007 and 89 from March 2013 to December 2014.

### PCR amplification and sequence analysis of *P. falciparumcrt* and *mdr*-*1* genes

Parasite DNA was isolated from 100 μl of blood sample using Takara DNA Blood Mini Kit following the manufacturer’s instructions (Takara, Kyoto, Japan). The extracted DNA was dissolved in TE buffer (10 mM Tris–HCl, 0.1 M EDTA, pH 8.0) and stored at −20 °C until use. Segments of the *pfcrt* and *pfmdr*-*1* genes spanning codons 72–76 (*pfcrt*) and codons 86, 184, 1034, 1042, and 1246 (*pfmdr*-*1*) were amplified as described protocol previously [22]. For *pfcrt* and *pfmdr*-*1* both the primary and nested amplifications were carried out in 25 μl of final volume with 10.0 μl of dH_2_O, 12.5 μl of *Taq* PCR Mast Mix (2.5 U), and 0.4 µM of forward and reverse primers, following the manufacturer’s instructions (Sangon Bio Inc., Shanghai, China) on a S1000 Thermal cycler (Bio-Rad, Hercules, USA). Primary amplification reactions were initiated with the addition of 2.0 μl of template genomic DNA prepared from the blood samples. For the nested PCR, 0.5 μl of primary PCR productions was used as a template. The amplified PCR products were separated on a 2.0 % agarose gel run with a 100 bp DNA ladder (Sangon Bio Inc., Shanghai, China). The nested PCR products were directly sequenced in both directions using an ABI PRISM3730 DNA sequencer (Sangon Bio Inc., Shanghai, China). The nucleotide and amino acid sequences of the *pfcrt* and *pfmdr*-*1* genes were compared with those of wild-type sequences of *pfcrt* (GenBank accession number KM288867) and *pfmdr*-*1* (GenBank accession number XM_001351751) using Clustal W of the BioEdit 7.0 and MEGA 4.0 programs.

### Statistical analysis

Statistical significance was determined using SPSS software (version 13.0). Mann–Whitney *U* test was used to compare in the frequencies of the mutations and alleles of the *pfcrt* and *pfmdr*-*1* in isolates collected between 2006–2007 and 2013–2014. *P* < 0.05 was considered statistically significant.

## Results

### Prevalence and patterns of mutant *pfcrt* gene in *P. falciparum* isolates

All blood samples (n = 207) were identified as *P. falciparum* mono-species infections after the thin blood smear examination. Because of low parasitaemia and/or poor DNA quality, some samples were not successfully amplified the *pfcrt* gene. Only 98 % of the *P. falciparum* isolates from Grande Comore Island (n = 202) could be amplified, including samples collected during 2006–2007 (n = 115) and 2013–2014 (n = 87). Mutations at codons C72S, M74I, N75E, and K76T of the *pfcrt* gene were detected in isolated from both 2006–2007 and 2013–2014 groups (Table 1). Mutant codon K76T was the most prevalent in both 2006–2007 and 2013–2014 groups, accounting for 72.3 and 19.5 % of the isolates examined, respectively. Over the course of 8 years (2006–2014), the number of non-synonymous mutations significantly decreased from 42.6 to 6.7 % at codon C72S (*P* < 0.01), from 39.1 to 14.9 % at codon M74I (*P* < 0.01), from 63.5 to 18.3 % at codon N75E (*P* < 0.01), and from 72.2 to 19.5 % at codon K76T (*P* < 0.01).

**Table 1:** Prevalence of *crt* and *mdr*-*1* mutations in *P. falciparum* isolates from 2006–2007 to 2013–2014 along Grande Comore island

Areas	Genes	Amino acid and genetic changes^a^	Number of isolates (%^b^)	
			2006–2007 (n = 115)	2013–2014 (n = 87)
Mitsoudje center hospital	*pfcrt*	C72**S** (TGT → **A**GT)	27 (23.5)	3 (3.4)**
		M74**I** (ATG → A**A**T)	23 (20.0)	5 (5.7)**
		N75**E** (AAT → **G**A**A**)	38 (33.0)	6 (6.9)**
		K76**T** (AAA → A**C**A)	41 (35.7)	5 (5.7)**
	*pfmdr*-*1*	N86**Y** (TGA → TG**T**)	45 (39.1)	25 (28.7)
		Y184**F** (ATA → AT**A)**	25 (21.7)	8 (9.2)**
		D1246**Y** (GAG → GA**T**)	12 (10.4)	6 (6.9)
National Malaria Center	*pfcrt*	C72**S** (TGT → **A**GT)	15 (13.0)	2 (2.3)**
		M74**I** (ATG → A**A**T)	13 (11.3)	4 (4.6)
		N75**E** (AAT → **G**A**A**)	19 (16.5)	6 (6.9)*
		K76**T** (AAA → A**C**A)	27 (23.5)	8 (9.2)**
	*pfmdr*-*1*	N86**Y** (TGA → TG**T**)	28 (24.3)	20 (23.0)
		Y184**F** (ATA → AT**A**)	21 (18.3)	12 (13.8)
		D1246**Y** (GAG → GA**T**)	10 (8.7)	2 (2.3)
Mitsamiouli Center Hospital	*pfcrt*	C72**S** (TGT → **A**GT)	7 (6.1)	1 (1.1)
		M74**I** (ATG → A**A**T)	9 (7.8)	4 (4.6)
		N75**E** (AAT → **G**A**A**)	16 (13.9)	4 (4.6)*
		K76**T** (AAA → A**C**A)	15 (13.0)	4 (4.6)*
	*pfmdr*-*1*	N86**Y** (TGA → TG**T**)	27 (23.5)	8 (9.2)**
		Y184**F** (ATA → AT**A**)	14 (12.2)	6 (6.9)
		D1246**Y** (GAG → GA**T**)	8 (6.8)	4 (4.6)
All the examined sites	*pfcrt*	C72**S** (TGT → **A**GT)	49 (42.6)	6 (6.9)**
		M74**I** (ATG → A**A**T)	45 (39.1)	13 (14.9)**
		N75**E** (AAT → **G**A**A**)	73 (63.5)	16 (18.3)**
		K76**T** (AAA → A**C**A)	83 (72.2)	17 (19.5)**
	*pfmdr*-*1*	N86**Y** (TGA → TG**T**)	100 (87.0)	35 (40.2)**
		Y184**F** (ATA → AT**A)**	60 (52.2)	26 (29.9)**
		D1246**Y** (GAG → GA**T**)	30 (26.1)	12 (13.8)*

^a^The mutated amino acids and nucleotides are indicated in bold type

^b^Statistically significant differences for comparison with isolates circulating in 2006–2007 from Grande Comore island (* P < 0.05; ** P < 0.01) using Mann–Whitney *U* test

Haplotype analysis of the *pfcrt* gene revealed that nine and six distinct allelic forms were detected in isolates from the 2006–2007 to 2013–2014 groups, respectively (Table 2). Of the nine allelic variants, the most prevalent were the quadruple-mutant allele (72S/74I/75E/76T) (26.1 %, 30/115) among 2006–2007 group isolates, followed by the WT allele (19.1 %, 22/115), triple mutant allele (74I/75E/76T) (13.0 %, 15/115), single mutant allele 76T (11.3 %, 13/115), and another triple mutant allele (72S/75E/76T) (10.4 %, 12/115), and a double mutant allele (75E/76T) (8.7 %, 10/115). The remaining three allelic variants were only detected in 13 (11.3 %) of *P. falciparum* isolates in the 2006–2007 group. Among the six allelic variants in the 2013–2014 group, WT allele was the most prevalent, accounting for 75.9 % (66/87). Except for the triple mutant allele (74I/75E/76T) with the frequency of 12.6 %, the remaining allelic variants were evenly distributed at low frequency (2.2 to 4.5 %) among isolates in the 2013–2014 group. Compared with the 2006–2007 group, the frequency of WT allele was significantly (*P* < 0.05) increased in those from 2013 to 2014 group, whereas the frequencies of single mutant allele 75E (*P* < 0.05) and 76T (*P* < 0.01), triple mutant allele 72S/75E/76T (*P* < 0.01), and quadruple-mutant allele 72S/74I/75E/76T (*P* < 0.01) were significantly decreased.

**Table 2:** Prevalence of single nucleotide polymorphisms and multi-mutated haplotypes in *crt* and *mdr*-*1* genes among Grande Comore *P. falciparum* isolates from different years

Genotypes^a^		Number of isolates (%)^b^		
		2006–2007 (n = 115)	2013–2014 (n = 87)	Total (n = 202)
*pfcrt*	Wild-type haplotype C_72_V_73_M_74_N_75_K_76_	22 (19.1)	66 (75.9)**	88 (43.6)
	Single-mutant haplotype **S**_72_V_73_M_74_N_75_K_76_	4 (3.4)	4 (4.5)	8 (4.0)
	Single-mutant haplotype C_72_V_73_M_74_ **E**_75_K_76_	6 (5.2)	0 (0)*	6 (3.0)
	Single-mutant haplotype C_72_V_73_M_74_N_75_ **T**_76_	13 (11.3)	2 (2.2)**	15 (7.4)
	Double-mutant haplotype **S**_72_V_73_M_74_N_75_ **T**_76_	3 (2.6)	0 (0)	3 (1.5)
	Double-mutant haplotype C_72_V_73_M_74_ **E**_75_ **T**_76_	10 (8.7)	2 (2.2)	12 (5.9)
	Triple- mutant haplotype **S**_72_V_73_M_74_ **E**_75_ **T**_76_	12 (10.4)	0 (0)**	12 (5.9)
	Triple-mutant haplotype C_72_V_73_ **I**_74_ **E**_75_ **T**_76_	15 (13.0)	11 (12.6)	26 (12.9)
	Quadruple-mutant haplotype **S**_72_V_73_ **I**_74_ **E**_75_ **T**_76_	30 (26.1)	2 (2.2)**	32 (15.9)
*pfmdr*-*1*	Wild-type haplotype N_86_Y_184_D_1246_	5 (4.3)	25 (28.7)**	30 (14.9)
	Single-mutant haplotype **Y**_86_Y_184_D_1246_	40 (34.8)	29 (33.3)	69 (34.2)
	Single-mutant haplotype N_86_ **F**_184_D_1246_	5 (4.3)	4 (4.6)	9 (4.5)
	Single-mutant haplotype N_86_Y_184_ **Y**_1246_	2 (1.7)	2 (2.3)	4 (2.0)
	Double-mutant haplotype **Y**_86_ **F**_184_D_1246_	35 (30.4)	13 (14.9)**	48 (23.8)
	Double-mutant haplotype **Y**_86_Y_184_ **Y**_1246_	8 (7.0)	5 (5.7)	13 (6.4)
	Double-mutant haplotype N_86_ **F**_184_ **Y**_1246_	3 (2.6)	3 (3.4)	6 (3.0)
	Triple-mutant haplotype **Y**_86_ **F**_184_ **Y**_1246_	17 (14.8)	6 (6.9)	23 (11.3)

^a^The mutated amino acids are indicated by bold type

^b^Statistically significant differences for comparison with isolates circulating in 2006–2007 from Grande Comore island (* P < 0.05; ** P < 0.01) using Mann–Whitney *U* test

### Prevalence and patterns of mutant *pfmdr*-*1* gene in *P. falciparum* isolates

The *pfmdr*-*1* segment was successfully amplified from 115 of 118 (97 %) and 87 of 89 (98 %) of the *P. falciparum* isolates collected from 2006–2007 to 2013–2014, respectively. Sequence analysis revealed point mutations at codons N86Y, Y184F, and D1246Y of the *pfmdr*-*1* gene in isolates collected in both 2006–2007 and 2013–2014 groups (Table 1). None of the investigated *P. falciparum* clinical isolates carried S1034C or N1042D substitution. Among the above three mutants, codon N86Y was the most prevalent, accounting for 87.0 %, followed by codons Y184F and D1246Y with frequency of 52.2 and 26.1 % in isolates from 2006 to 2007 group, respectively. Similarly, the N86Y was the most prevalent in the 2013–2014 group, accounting for 40.2 %, followed by codons Y184F and D1246Y with frequency of 29.8 and 13.8 %, respectively. The frequencies of mutations at codons N86Y (*P* < 0.01), Y184F (*P* < 0.01), and D1246Y (*P* < 0.05) were significantly decreased in the 2013–2014 group compared with those in the 2006–2007 group, respectively.

Haplotype analysis of *pfmdr*-*1* gene of the samples revealed eight distinct allelic forms (Table 2), including the wild type allele, three single-mutant alleles (86Y, 184F, and 1246Y), three double-mutant alleles (86Y/184F, 86Y/1246Y, and 184F/1246Y), and one triple mutant allele (86Y/1246Y/1246Y). Of the eight allelic variants, the most prevalent allelic variant was single-mutant allele 86Y in isolates from both 2006–2007 (34.8 %, 40/115) and 2013–2014 (33.3 %, 29/87) groups, followed by double-mutant allele 86Y/184F in isolates from 2006 to 2007 (30.4 %, 35/115) and wild type allele in isolates from 2013 to 2014 group (28.7 %, 25/115). Compared with the 2006–2007 group, the frequency of WT allele (changed from 4.3 to 28.7 %) was significantly (*P* < 0.01) increased in those from 2013 to 2014 group, whereas the frequency of double-mutant allele (86Y/184F) was significantly decreased (from 30.4 to 14.9 %; *P* < 0.01).

## Discussion

In Comoros, with the official ban of CQ in 2004, CQ has now been out of use for almost 12 years although self-medication might have continued for a few years after the ban. The dramatic reduction of annual malaria cases in Grande Comore may be mainly due to ACT-based MDA regimens and other malaria control interventions. However, the emergence and spread of artemisinin-resistant *P. falciparum* parasites in Southeast Asia [8–10] and the increased K13-propeller gene diversity among *P. falciparum* populations from 2006 to 2014 in Grande Comore (a ~20 % increment) [11, 12] may open up a new challenge in the ACT efficacy in the future and call for monitoring the changes in parasite susceptibility to CQ and other anti-malarial drugs. The issue as to whether or not CQ-susceptible strains may be returned in malaria-endemic regions after the withdrawal of CQ selective pressure is of great interest for malaria control. This study assessed the evolution of CQR by investigating the temporal variations of *pfcrt* and *pfmdr*-*1* gene polymorphisms in *P. falciparum* isolates collected from Grande Comore for two different periods (2006–2007 and 2013–2014). Due to the dramatic reduction of annual malaria cases in Grande Comore since 2013, the number of *P. falciparum* isolates collected from 2013 to 2014 (n = 89) was smaller than those from 2006 to 2007 (n = 118). Our data showed that the frequencies of mutant alleles *pfcrt* and *pfmdr*-*1* significantly decreased in isolates from 2013 to 2014 when compared with those from 2006 to 2007, suggesting that CQS *P. falciparum* populations have returned to Grande Comore after the withdrawal of CQ.

The long history of CQ use has exposed *P. falciparum* to the drug pressure continually in Comoros. Clinical failure after standard CQ treatment was initially reported in a case of *P. falciparum* malaria in Comoros in 1980 [4]. A trend of gradual increment of CQ clinical failure rates (from 31 to 90 %) had been documented in this malaria-endemic area between 1990 and 2004 subsequently [7, 26, 27]. Sets of SNPs in the *pfcrt* codons 72, 74, 75, and 76 were associated with CQR in *P. falciparum* from Southeast Asia, Africa and South America [14, 32]. The *pfcrt* K76T mutation has been considered as the most reliable molecular marker of CQR among the various mutations identified [14, 32]. In the present study, the high frequency (72.2 %) of *pfcrt* K76T was observed in *P. falciparum* isolates from 2006 to 2007. Our data are consistent with those in other reports, where 45–80 % of Comoros *P. falciparum* isolates collected in 2006–2007 had the *pfcrt* K76T mutation [29–31]. We observed an increasing trend in the prevalence of the *pfcrt* K76 wild type allele (19.1 % in 2006–2007; and 75.9 % in 2013–2014) and a decreasing trend for *pfcrt* K76T mutation (72.2 % in 2006–2007; and 19.5 % in 2013–2014). The decreasing trend for *pfcrt* K76T mutation in our study is consistent with several previous reports from other malaria endemic regions. In Ghana, a decrease in frequency of the *pfcrt* K76T mutation (from 88 to 56 % for 2005–2010) was observed [33]. In Malawi [15] and Kenya [19], decreases from 85 to 13 % between the years of 1993 and 2000 and from 95 to 60 % between 1993 and 2006 were reported, respectively. Similar decreases in mutant *pfcrt* were reported in many other regions, including Senegal [34], Mozambique [35], Tanzania [23], and Madagascar [36, 37]. In another in vivo study*, P. falciparum* parasites carrying the CQS *pfcrt* K76 allele was selected after treatment with AL [38]. The observation of increased CQS *pfcrt* K76 allele after AL use is consistent with our results; discontinue of CQ use (or CQ related drugs such as amodiaquine) allows the return of parasites with wild type *pfcrt* allele. Thus, the decreasing trend for *pfcrt* K76T mutation observed in our study may highlight a benefit of using AP or AL in an area with a high prevalence of CQ-resistant *P. falciparum* malaria such as Grande Comoros, but not artemether-lumefantrine-amodiaquine (AQ) because AQ (with amodiaquine) was shown to select for *pfcrt* 76T allele and *pfmdr1* YYY haplotype [39]. In contrast, high frequencies of the *pfcrt* K76T mutant were detected in some countries despite the withdrawal of CQ for many years, including Gabon [17] and Benin [24]. The decreases in mutant *pfcrt* alleles after withdraw of CQ use is likely due to fitness cost of the *pfcrt* mutations, and the persistence of high frequencies of the mutant alleles in some parasite populations is because of compensatory changes in the parasite genome that allow the parasite to overcome the negative effects of drug resistant mutations. For example, in a study of gene expression in *pfcrt* mutants, CQ treated *pfcrt* mutants were found to have significant enrichment in glycerol and polyol metabolic processes and iron/cation transport activities [40]. Compensatory mutations have been widely reported in other microorganisms [41].

The mutant *pfcrt* SVMNT haplotype, which is mostly associated with amodiaquine resistance and lower level of CQR compared to CVIET [42], had been reported mostly in Southeast Asia (India and Laos) [43–45] and South America [46, 47], but is still very rare in Africa [48]. In the present study, the mutant *pfcrt* SVMNT (2.6 %, 3/195) observed in the *P. falciparum* isolates examined was identical to that of CQR isolates identified in Ethiopia [22] and Tanzania [48]. Therefore, results from the current study suggest that *P. falciparum* populations with the mutant *pfcrt* SVMNT allele may have a global distribution now.

Although *pfmdr*-*1* haplotype alone does not determine the level of CQ resistance, the mutations at codons N86Y, S1034C, N1042D, and D1246Y in *pfmdr*-*1* gene are related to reduced sensitivity to CQ [49]. It was reported that CQR *P. falciparum* populations were introduced into Madagascar from Comoros islands [29], and in vivo resistance to CQ in Madagascar that was not associated with the *pfcrt* 76T mutation, but with the mutation *pfmdr*-*1* 86Y and perhaps with other yet unknown mechanisms [49, 50]. In the present study, none of the *P. falciparum* clinical isolates collected in 2006–2007 and 2013–2014 carried S1034C and N1042D substitutions, which was similar to those reported from Madagascar [30]. In the present study, three mutations at codons N86Y (87.0 %), Y184F (52.2 %), and D1246Y (26.1 %) were detected in the 2006–2007 group isolates. The results from this study are similar to other previous reports in Grande Comore, where 97.4 and 77.5 % of parasite isolates collected in 2006 had mutations at codons N86Y and D1246Y, respectively, followed by only 1.8 % of isolates with *pfmdr*-*1* wild type gene [29, 30]. In the present study, over the course of 8 years (2006–2014), the frequencies of mutations in *pfmdr*-*1* gene dramatically changed from 87.0 to 40.2 % (at codon N86Y, *P* < 0.01) and from 26.1 to 13.8 % (at codon D1246Y, *P* < 0.05). Our data are in line with those of a report describing the change in frequency of *pfmdr*-*1* gene mutations in Madagascar isolates [30, 37], in which the *pfmdr*-*1*mutation N86Y was reduced from 50 to 11 % 6 years after the withdrawal of CQ. Similarly, with the prevalence of *pfmdr*-*1* N86Y reaching a peak in 2000 (78 %), there was a highly significant decline in prevalence of the *pfmdr*-*1* N86Y allele in 2008 in Gambia [18]. Therefore, the observations from this study suggested that an increasing trend in *P. falciparum* susceptibility to CQ may exist in Grande Comore currently. However, this observation showed that over the course of 8 years (2006–2014), the frequencies of mutations in *pfmdr*-*1* gene at codon Y184F dramatically changed from 52.2 to 30.0 % (*P* < 0.01) in the present study. The observation was in contrast to other reports from Kenya [39], Uganda [51], and Sudan [52] where the *pfmdr*-*1* 184F allele has been previously associated with in vivo selection by AQ or AL.

In the present study, the decline in prevalence of *pfmdr*-*1* gene mutations (~24 %) was lower than those in *pfcrt* gene mutations (~67 %) among Grande Comore isolates over the course of 8 years (2006**–**2014). The observation is line with other reports describing *mdr*-*1* and *crt* polymorphisms in *P. falciparum* isolates collected from Malawi, in which ~30 or ~70 % decline was observed in the change of prevalence of mutant *pfmdr*-*1 or pfcrt* among *P. falciparum* isolates collected from 1992 to 2000, respectively [15]. These observations suggest that *pfmdr*-*1* mutations may be less deleterious to parasite fitness than the *pfcrt* mutations [13, 14], even though *pfmdr*-*1* mutations can modulate the level of CQ resistance.

In the present study, the rapid shift in *P. falciparum* from CQR to CQS suggests that the replacement of CQ with ACT for the treatment of *P. falciparum* may eventually result in the significant decline of *pfcrt* and *pfmdr*-*1* mutations or CQ-resistant strains in Grande Comore isolates. It was reported that the return of CQS was the result of re-expansion of the susceptible parasites, but not back mutations in a formerly resistant parasite or a new selective sweep in Africa [20] where the malaria transmission rates are higher and naturally immune individuals are more common compared with those in Southeast Asia. Whether or not the CQS resurgence is due to the expansion of surviving CQS reservoir populations or back-mutations in the CQR allele in Grande Comore can not be inferred from our results.

## Conclusions

Results from the current study showed that the prevalence and patterns of mutant *pfcrt* and *pfmdr*-*1* dramatically decreased in Grande Comore isolates over the course of 8 years (2006–2014). Although the data related to the in vivo or in vitro efficacy of CQ of the parasites was not collected in this study, the present data suggest that the CQS *P. falciparum* parasites have returned in Grande Comore to some extent after the official withdrawal of CQ. The data presented here will be useful for developing and updating anti-malarial policies in Grande Comore.

## Abbreviations

CQ: chloroquine
CQS: CQ-sensitive
CQR: CQ-resistance
*Pfcrt*: *P. falciparum* CQ resistant transporter
*Pfmdr*-*1*: *P. falciparum multiple drug resistance 1*
ACT: artemisinin-based combined therapy
AL: artemether-lumefantrine
AP: artemisinin-piperaquine
AQ: artemether-amodiaquine
